# Amyloid Assembly Endows Gad m 1 with Biomineralization Properties

**DOI:** 10.3390/biom8010013

**Published:** 2018-03-20

**Authors:** Milagros Castellanos, Almudena Torres-Pardo, Rosa Rodríguez-Pérez, María Gasset

**Affiliations:** 1 IMDEA Nanoscience, 28049 Madrid, Spain; milagros.castellanos@imdea.org; 2 Departamento de Química Inorgánica, Facultad de Químicas, Universidad Complutense, 28040 Madrid, Spain; atorresp@quim.ucm.es; 3 Instituto de Investigación Hospital Universitario La Paz (IdiPaz), 28046 Madrid, Spain; rosa.rodriguez@idipaz.es; 4 Instituto Química-Física “Rocasolano”, Consejo Superior de Investigaciones Científicas, 28006 Madrid, Spain

**Keywords:** amyloids, Gad m 1, EF-hand motif, calcium carbonate precipitation, calcite

## Abstract

Acid proteins capable of nucleating Ca^2+^ and displaying aggregation capacity play key roles in the formation of calcium carbonate biominerals. The helix-loop helix EF-hands are the most common Ca^2+^-binding motifs in proteins. Calcium is bound by the loop region. These motifs are found in many proteins that are regulated by calcium. Gad m 1, an Atlantic cod β-parvalbumin isoform, is a monomeric EF-hand protein that acts as a Ca^2+^ buffer in fish muscle; the neutral and acid apo-forms of this protein can form amyloids. Since Ca^2+^-nucleating proteins have a propensity to form extended β-strand structures, we wondered whether amyloid assemblies of an EF-hand protein were able to influence calcium carbonate crystallization in vitro. Here, we used the Gad m 1 chain as a model to generate monomeric and amyloid assemblies and to analyze their effect on calcite formation in vitro. We found that only amyloid assemblies alter calcite morphology.

## 1. Introduction

Calcium carbonate biominerals are the most abundant natural biocomposites; these biominerals are used for shell formation and for balancing systems [1,2]. Acid proteins play major roles in the nucleation, growth and morphology of carbonate crystals by modulating Ca^2+^ condensation [3,4,5,6,7,8]. These proteins also share an oligomerization propensity involving extended β-strand structures, suggesting that amyloid protein assemblies with acidic regions could acquire biomineralization properties, such as the ability to modulate calcium carbonate crystallization [3,9,10,11].

Amyloid aggregates share an intermolecular cross-β sheet motif as a scaffold and a variable morphology. These assemblies are usually unbranched fibrils with widths of 8–30 nm and micrometer lengths that result from intertwisting of protofilaments made of pairs of β-sheets [12,13]. However, the shape and topology of the aggregate can be modified by changing the sequence and the conditions used for their formation [14,15]. Despite their variations in size and shape, all amyloids are characterized by structural repetitiveness and the resulting interactions [12]. For instance, Pmel amyloids enable the organized binding of melanin precursors and their efficient covalent polymerization into mature melanin [16,17]. In other cases, the amyloid fold of the segment leads to the presence of novel binding sites absent in the monomer precursor, as in the case of Zn^2+^ and the assembled Ac-IHVHLQI-CONH_2_ peptide [18,19].

Among distinct cation sites, the EF-hand is a widespread Ca^2+^-binding motif. An EF-hand is usually 30 residues long and folds into a helix-loop-helix structure in which the canonical 12-residue interhelical acid loop coordinates one cation with pentagonal bipyramidal symmetry [9]. This motif often occurs in pairs that result in the EF-lobe [20]. Gad m 1, an Atlantic cod β-parvalbumin isoform with allergenic properties, represents a model of a minimal EF-lobe protein [21,22,23]. The Gad m 1 chain contains three tandemly arrayed EF-hands, of which only the two C-terminal motifs named CD and EF bind Ca^2+^. In the cation-bound form, Gad m 1 displays a highly stable monomeric helical globular fold [24]. In contrast, removal of bound Ca^2+^ at high protein concentrations triggers Gad m 1 amyloid aggregation through the regions forming helix B and helix D in the globular fold [25,26,27]. Once formed, amyloids partially dissociate into monomers upon Ca^2+^ addition [25].

Here we used a protein that is not involved in biocomposites, cod parvalbumin protein Gad m 1, to analyze the effect of its aggregation state on calcium carbonate precipitation. We found that amyloid assemblies, but not monomers, perturbed calcite crystallization from the conventional rhombohedral habit to a sheaf-like morphology.

## 2. Results

**Figure 1:**
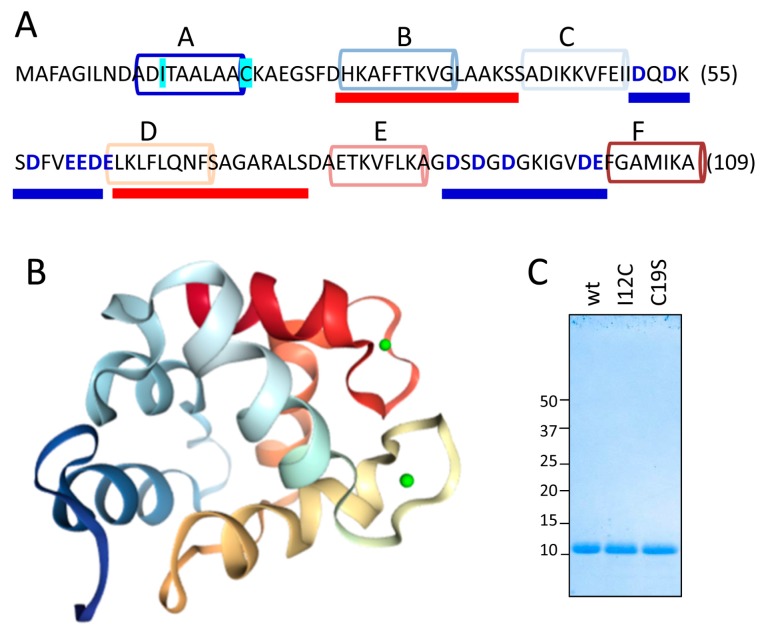
Structural features of the Gad m 1 chain. (**A**) Gad m 1 sequence and its dual folding signatures. Helical segments A–F forming the helix-loop-helix (AB, CD and EF) of EF-hands are depicted by colored cylinders. Acid loops binding Ca^2+^ and joining C and D and E and F helices are underlined by a thick blue line. Regions forming the aggregated core of the amyloid fold are underlined by a thick red line. Residues used for mutant generation (I12C and C19S) are outlined in cyan. (**B**) Three-dimensional structure of Ca^2+^-bound Gad m 1 (PDB 2MXY). Helical segments are depicted using the color code used in panel A, and cations are depicted as green spheres. (**C**) Sodium dodecyl sulfate polyacrylamide gel electrophoresis (SDS-PAGE) gel of the recombinant Gad m 1 wild type (wt), I12C and C19S chains obtained after cleavage of the His-tag.

Figure 1A,B show the sequence and the structural features, respectively, of the Gad m 1 chain (UniProtKB A5I874, PDB 2MBX). The 109-residue chain is organized into three consecutive EF-hand motifs (AB, CD, and EF); only CD and EF contain acid loops with functional Ca^2+^ binding. Regions B and D contain amyloid-promoting sequences, which are defined as sequences with alternating amyloid-forming and acid segments in the aggregated state; these sequences can function as Ca^2+^-nucleating centers for biomineralization events [26,27]. However, amyloids are polymorphic entities; studying their distinct aggregates could shed light on the features of the Ca^2+^-nucleating process. As previously reported, aggregation is regulated by the region A sequence [25,26]. This segment is in the C-terminal and flanked by a cysteine residue (C19), as in all fish β-parvalbumins, but the segment lacks the cysteine residue (C12) in its N-terminal region that characterizes some isoforms, suggesting that the Cys pattern plays a role in the properties of the amyloid state of the protein [25,26,28]. Therefore, Gad m 1 chains with differing Cys patterns in region A may result in distinct amyloid assemblies and Ca^2+^-nucleating activities.

**Figure 2:**
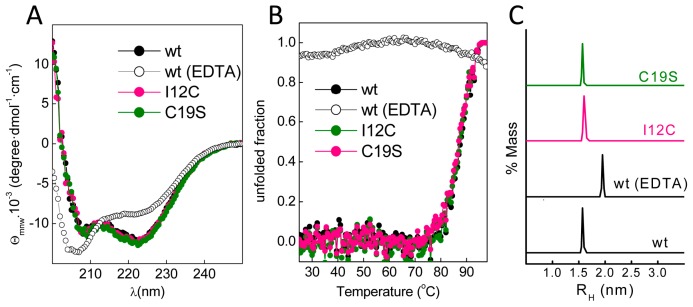
Conformational features of the Ca^2+^-bound Gad m 1 wt and mutant chains. (**A**) Far-ultraviolet (UV) CD spectra of Ca^2+^-bound Gad m 1 chains depicting their helical fold. (**B**) Thermal denaturation of Ca^2+^-bound Gad m 1 chains displaying the cooperativity and stability of their folds. Denaturation curves were obtained from the changes in molar ellipticity at 222 nm that occurred upon heating. (**C**) Hydrodynamic radius of Gad m 1 chains derived from DLS measurements with an average polydispersity of 5–10%. The label wt ethylenediaminetetraacetic acid (EDTA) corresponds to Gad m 1 wt treated with 5 mM EDTA, which was included as a folding control. R_H_: hydrodynamic radii, θmrw: mean residue weight ellipticity.

With this information in mind, we produced Gad m 1 wild type (wt), I12C and C19S recombinant proteins that yielded 11 kDa bands when analyzed by sodium dodecyl sulfate polyacrylamide gel electrophoresis (SDS-PAGE) (Figure 1C). The far-ultraviolet (UV) CD spectra of all proteins prepared in 5 mM CaCl_2_ had minima at 222 and 208 nm; this feature is characteristic of α-helix rich structures (Figure 2A). These spectral features are lost in Gad m 1 wt upon removal of the bound cation, indicating that in the presence of Ca^2+^, the chains exhibited the native secondary structure. To confirm the existence of tertiary contacts, we used thermal denaturation experiments due to the absence of intrinsic chromophores. Figure 2B shows that the unfolding of the Ca^2+^-bound proteins measured by the changes in the ellipticity at 222 nm with temperature proceeds cooperatively with a half-live (T_1/2_) of 88 ± 0.5 °C. These unfolding features disappeared in control measurements using ethylenediaminetetraacetic acid (EDTA)-treated Gad m 1 wt, indicating that all Ca^2+^-bound proteins had a compact fold. Furthermore, dynamic light scattering (DLS) was used to characterize the hydrodynamic properties of the proteins. Figure 2C shows that the Ca^2+^-bound wt, I12C and C19S proteins had a hydrodynamic radius of 1.7 ± 0.1 nm with 5–10% polydispersity and that this value increased to 2.0 ± 0.1 nm upon cation removal. Taken together, these data indicate that all Ca^2+^-bound chains displayed the characteristic monomeric α-helical fold of fish β-parvalbumins and agree with previous reports [23,25,26].

**Figure 3:**
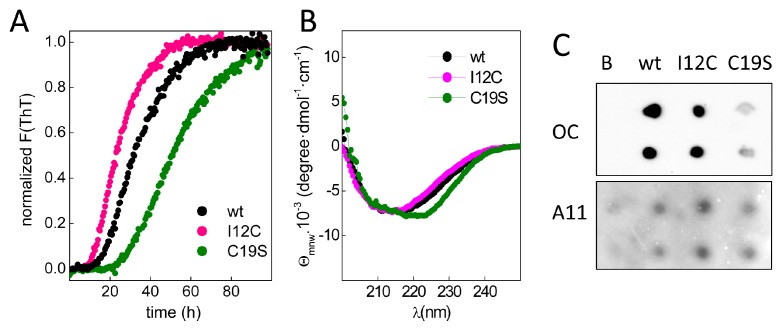
Amyloid aggregation of Gad m 1 chains. (**A**) Kinetics of amyloid aggregation monitored by thioflavin T (ThT) fluorescence. (**B**) CD spectra of Gad m 1 aggregates isolated by ultracentrifugation. (**C**) Dot-blot analysis of the recognition of the aggregation reaction products by anti-amyloid fibril (OC) and anti-amyloid oligomer (A11) antibodies. The label B corresponds to the Ca^2+^-bound Gad m 1 wt monomer.

To study chain aggregation, 2 mg/mL protein solutions were incubated in the presence of EDTA at 37 °C, and the fluorescence changes in samples with added thioflavin T (ThT) were monitored over time. Figure 3A shows that upon incubation, all chains exhibited a similar increase in the fluorescence intensity but had differences in the lag phases, which were inversely correlated with the number of Cys residues in region A of the chain. Moreover, the increase in ThT fluorescence was accompanied by the formation of insoluble aggregates with β-sheet-rich secondary structures (Figure 3B) and epitopes for anti-amyloid fibril (OC) and anti-amyloid oligomer (A11) antibodies (Figure 3C), agreeing with the amyloid signature of the aggregates. Despite the general trend of these features, the differential spectral properties and the reduced OC recognition of C19S suggest potential distinct amyloid states.

**Figure 4:**
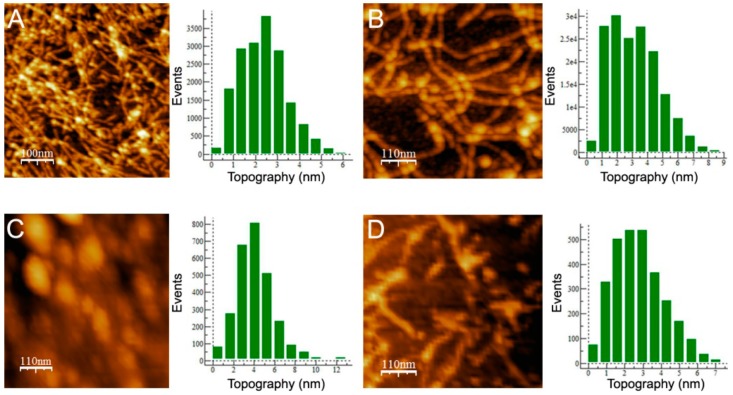
Atomic force micrographs of Gad m 1 amyloid assemblies. Images of the aggregation products of Gad m 1 (**A**) wt, (**B**) I12C, (**C**) C19S and (**D**) wt in presence of tris(2-carboxyethyl)phosphine hydrochloride (TCEP). Height histograms correspond to the full images.

To gain insight into the aggregate shapes, we used atomic force microscopy (AFM). Figure 4A shows that wt aggregates were mainly thin protofibrillar assemblies with a 2.5 nm average height, approximately 12 nm width and variable lengths greater than 300 nm, in agreement with previous work [25]. I12C aggregates also appear as protofibrils, but these protofibrils have an average height of 3 nm and a larger width (25 nm) (Figure 4B). In this case, the abundance of crosslinks among protofibrils and their curviness impede the determination of a characteristic length. Comparison of both major features of these two aggregates, which display similarities in secondary structure and OC reactivity, indicates that the presence of C12 alters the morphology of the protofibrillar assembly by increasing its dimensions. In contrast, C19S aggregates appear as rod-like nanoplatelets of 4 nm height, 50 nm widths and 150 nm length (Figure 4C). Comparing the morphologies of wt and C19S aggregates indicates that C19 is a key residue for either the formation or the stabilization of the protofibrillar assembly. Indeed, treatment of wt protofibrils under the reducing conditions provided by 10 mM tris(2-carboxyethyl)phosphine hydrochloride (TCEP) caused the exposure of protofibrils and the introduction of fuzzy nanoparticles (Figure 4D). Therefore, chains with disulfide bonding capacity in region A form protofibrillar assemblies, and their thickness is a function of the number of potential disulfide bonds. In contrast, Cys-free chains form rod-like nanoplatelets.

**Figure 5:**
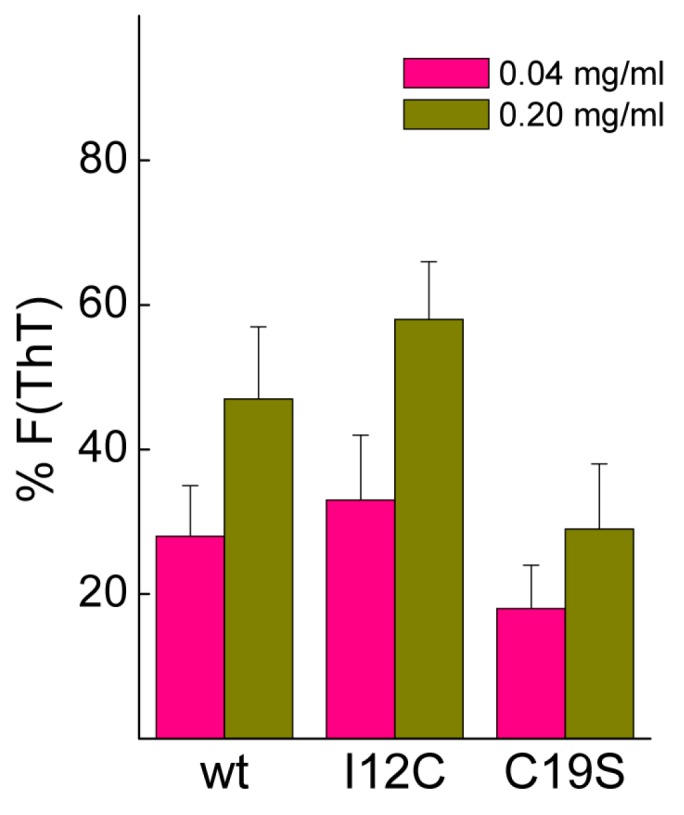
Stability of amyloid aggregates under calcium carbonate crystallization conditions. Amyloid dissociation was determined as the percentage of thioflavin T (ThT) fluorescence remaining after 20 h incubation in 0.1 M CaCl_2_.

Biomineralization activity assays conventionally involve the dilution of proteins in 0.1 M CaCl_2_, conditions that may cause dissociation of Gad m 1 aggregates [25]. To assess their stability, protein aggregates at final concentrations of 0.04 and 0.2 mg/mL were placed in 0.1 M CaCl_2_ for 20 h, and the changes in the fluorescence of the amyloid probe ThT were determined. Figure 5 shows that after 20 h, the fluorescence intensity is significantly reduced, and the decrease is inversely correlated with the protein concentration. Despite the lack of significance among the differences, the data suggested the protofibrillar amyloids formed by chains containing Cys residues (wt and I12C) better resisted the solubilizing action of Ca^2+^ than did the nanoplatelets formed from the C19S chain.

**Figure 6:**
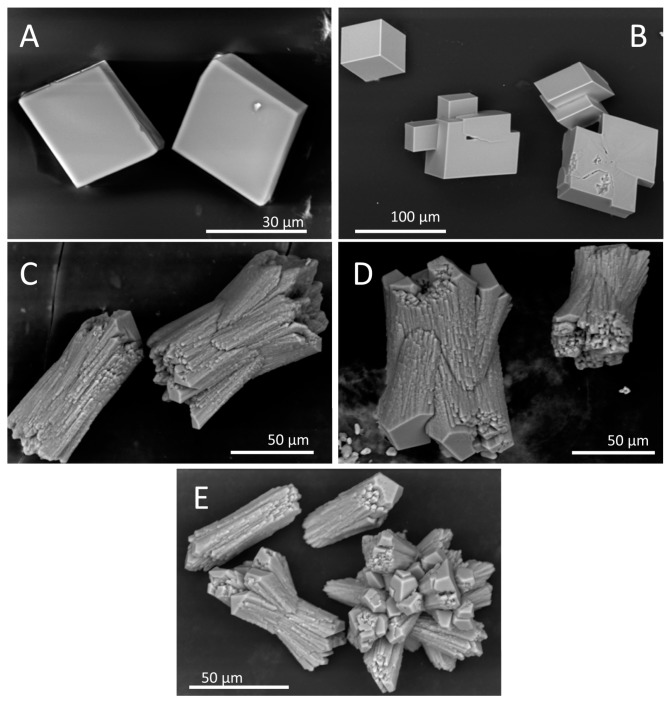
Scanning electron microscopy (SEM) images of the calcium carbonate precipitates obtained in vitro. Precipitates were obtained in the (**A**) absence and presence of Gad m 1 (**B**) wt monomer, (**C**) wt amyloids, (**D**) I12C amyloids and (**E**) C19S amyloids. The protein concentration was 0.2 mg/mL.

To investigate the influence of amyloids on calcium carbonate precipitation, aggregates were placed under the previous conditions, and the reaction was enabled by controlled diffusion of CO_2_ from the decomposition of ammonium carbonate. As a control for the non-polymerized and Ca^2+^ pre-complexed protein form, we used Ca^2+^-bound Gad m 1 wt monomers at similar concentrations [24,25]. The representative crystal structures that formed were visualized by scanning electron microscopy (SEM) (Figure 6). In the absence of protein, the common rhombohedral form of calcite crystals was observed (Figure 6A). Reactions performed in the presence of Gad m 1 monomers yielded rhombohedral calcite crystals with smooth surfaces (Figure 6B), indicating that the globular fold of Gad m 1 had a weak influence on calcite morphology. In other words, the acid loops of the folded EF-lobe did not participate in the crystallization process. In contrast, the presence of Gad m 1 wt, I12C and C19S amyloids caused the precipitation of distinct cylindrical and sheaf-like crystals (Figure 6C–E). For each chain assembly, the changes were proportional to the protein concentration used (data not shown). Importantly, despite the global shape similarities, each of the aggregates caused specific morphological signatures. For instance, Gad m 1 wt protofibrils yielded 75 µm long cylindrical and sheaf-like crystals with 45–50 µm diameters with regular capped surfaces (Figure 6C). The crystals obtained from I12C protofibrils mostly had sheaf-like morphologies with a 125 µm length, 70 µm diameter, and regular capped surfaces (Figure 6D). Nanoplatelets of the C19S mutant yielded smaller crystals consisting of a mixture of cylinders (25 and 50 µm for the length and diameter, respectively) and tightly bound sheaves (Figure 6E).

**Figure 7:**
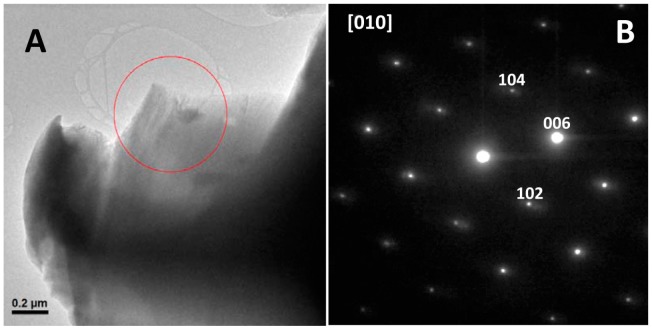
Representative (**A**) Transmission electron microscopy (TEM) image and the corresponding (**B**) Selected area electron diffraction (SAED) pattern from the marked area of the crystal along the [010] projection of the powder calcium carbonate precipitates obtained in the presence of Gad m 1 wt and mutant amyloids.

These SEM results show that Gad m 1 amyloids caused a general morphology change in calcium carbonate crystals but do not indicate whether the morphology alteration also involves an internal structure change from the structure of calcite to the structure of its polymorph aragonite [1,2,3]. To probe the internal structure, we obtained their selected area electron diffraction (SAED) patterns by transmission electron microscopy (TEM). The low magnification image and the corresponding SAED diffraction pattern of a representative crystal of all analyzed samples is shown in Figure 7. All diffraction maxima can be indexed on the [010] zone axis of the rhombohedral calcite unit cell [29]. Therefore, taken together, these results indicate that the amyloid assembly of Gad m 1 chains allows acid loops to nucleate Ca^2+^ and switch calcite crystallization from a rhombohedral to a sheaf-like morphology.

## 3. Discussion

Recent research showed that amyloid aggregates constitute a basic scaffold for their functional exploitation in material science such as in the generation biominerals. In the present study, we asked whether amyloid assemblies of a protein with acid regions can acquire biomineralization properties, such as modulation of calcium carbonate crystallization. To test this hypothesis, we used Gad m 1. This fish β-parvalbumin can transition from a three EF-hand globular fold to a β-sheet rich amyloid aggregate via a change in the Ca^2+^ binding to the C-terminal acid loops. Gad m 1 amyloids are supported by the assembly of the two segments, and these amyloids had two acid regions that were free from structural constraint. The relative chain location of both types of segments defines a design characterized by alternating amyloid-promoting and acid regions; this design differs from other self-assembly peptides used previously in Ca^2+^-nucleating reactions, such as the P11-4 peptide (Ace-QQRFEWEFEQQ-NH2) [30]. Our study revealed that the amyloids formed with distinct Gad m 1 chains influence calcium carbonate precipitation, causing the appearance of calcite crystals with different cylindrical and sheaf-like morphologies, which differ from the characteristic rhombohedral form of calcite. Importantly, the use of distinct aggregates resulting from the assembly of mutant chains led to crystals that had specific characteristics.

Apart from the well-known Ca^2+^-buffering physiological role of the Gad m 1 globular fold, the ability to form amyloid aggregates has recently been associated with augmented immunoglobulin E (IgE) recognition in food allergies [21,22,23,24,25]. Amyloids are polymorphic structures, and their shape and properties can be modulated by varying the physical formation conditions and the covalent structure of the chain used [12,13]. Targeting the disulfide bonding capacity of the regulatory region distal to the amyloid cores has allowed protofibrillar and rod-like amyloid aggregates of the Gad m 1 chain. Protofibrillar assemblies appear as a result of disulfide bonding of the N-terminal region, and the assemblies can be converted into rod-like shapes after their reduction. Since rod-like nanoplatelets dissociated easier than did protofibrils, it is tempting to speculate that any in vivo-formed amyloid might be strictly controlled by redox processes.

In contract to the globular fold, all Gad m 1 amyloids modulate calcium carbonate precipitation. This property makes them attractive environmentally friendly tools for their use in decayed-stone protection and CO_2_ deposition devices. These amyloids mainly trap CO_2_ using bound Ca^2+^, whereas other amyloid-based traps uniquely bind CO_2_ via carbamate formation with their Lys residues [31]. The proven function of both CO_2_-trapping mechanisms supports future designs of novel hybrid amyloids with amplified deposition efficiency using a Lys-based core and appropriate acidic flanking residues.

Calcite crystallization from calcium carbonate precipitates occurs in layers, and differences in the relative growth rates in the distinct axes impact the shape and morphology of crystals [32,33]. Gad m 1 amyloid mutants with differing Cys contents showed a common pattern of modification in the relative growth rates that yielded morphologies distinct from those previously reported for both proteins that are associated with biocomposites and those that are not [2,3,4,5,6,7,8,9,10,11,32,33,34,35]. Despite the common sheaf-like morphology, the crystals displayed chain-dependent features that can help impart amyloid differences. For instance, the crystal size was larger in precipitation reactions using protofibrils (produced by Gad m 1 wt and the I12C mutant) than in those using the rod-like nanoplatelets formed by C19S. However, the diameter of the crystals obtained in the presence of protofibrils varied according to the assembly width. Importantly, Gad m 1 monomers in which the acid loops define the Ca^2+^-binding sites have a weak influence on calcite crystallization, underscoring the role of surface charges such as those reported for the aggregates formed by the egg shell-forming ovocleidin-17 [34].

Calcium carbonate particles are appealing due to their applications in the removal of heavy metal ions from waters, the improvement of the mechanical properties of foam, and the generation of ultrasonic-sensitive drug delivery devices [36]. Although much work is needed, our results provide a proof of concept for the functional exploitation of a non-physiological amyloid in the field of calcium carbonate-based materials.

## 4. Materials and Methods

### 4.1. Production of Gad m 1 wt and Mutant Chains

Gad m 1 (UniProtKB A5I874) was produced from a pET15b construct that was previously described [25]. Mutants I12C and C19S were generated using QuickChange protocols and the oligos (forward only) 5′-CGATGCGGATTGCACCGCGGCG-3′ and 5′-GCGCTGGCGGCGAGCAAAGCGGAAGGC-3′. Changes were verified by sequencing. All proteins were produced in *Escherichia coli* BL21 (DE3), isolated and purified as described previously [26]. The N-terminal His-tags were removed using a Thrombin CleanCleave Kit according to the manufacturer’s indications (Sigma-Aldrich, St. Louis, MO, USA). Before their use, protein solutions were extensively dialyzed against 5 mM of 4-(2-hydroxyethyl)-1-piperazineethanesulfonic acid (HEPES) (pH 7.5) containing 0.1 mM CaCl_2_, concentrated using 10 kDa pore size Amicon Ultra-15 filter (Merck, Darmstadt, Germany) units and centrifuged at 16,000× *g* for 20 min at 4 °C to remove aggregates. Protein concentrations were determined using the Bradford protein assay (BioRad, Hercules, CA, USA) [26]. SDS-PAGE was performed using MiniProtean TGX precast AnykD gels and Coomassie blue staining (BioRad).

### 4.2. Circular Dichroism Spectroscopy

Circular dichroism measurements were performed in a Jasco J-820 spectropolarimeter using 0.1 cm cuvettes and a thermostated cell holder. Spectra were recorded with 20 μM protein solutions in 10 mM Tris-HCl (pH 7.5) containing 35 mM NaCl and 5 mM CaCl_2_. For thermal denaturation experiments, Tris-HCl was replaced with HEPES-HCl, and the ellipticity changes at 222 nm that occurred upon heating from 15 °C to 90 °C at a 1 °C/min heating rate were monitored. Both spectra and denaturation curves were analyzed as previously described [25,26].

### 4.3. Dynamic Light Scattering

Dynamic light scattering measurements were performed in a DynaPro spectroscatter (Wyatt Technology, Dernbach, Germany) at 25 °C using a thermostated 30 μL quartz cuvette and samples of 180 μM (Ca^2+^-bound protein) and 60 μM (Ca^2+^-free protein) protein concentrations. The hydrodynamic radii (R_H_) and mass proportions (%) of the species were determined from the average of 20 acquisitions using a cumulative fit as previously described [25]. Measurements were performed in duplicate using two different protein batches.

### 4.4. Amyloid Formation and Stability

Amyloid aggregates were formed by incubating proteins (2 mg/mL) in 25 mM Tris-HCl (pH 7.5) containing 50 mM NaCl and 4 mM EDTA for 120 h at 37 °C. Briefly, after aggregates were removed by centrifugation, protein stock solutions were diluted with 25 mM Tris-HCl (pH 7.5) containing 50 mM NaCl and 4 mM EDTA and supplemented with or without 10 μM ThT (Calbiochem, Darmstadt, Germany) for fluorescence experiments. Reactions were initiated by placing the sealed 96-well plate (0.150 mL solution/well) at 37 °C in a POLARstar (BMG Labtech, Ortenberg, Germany) microplate reader. The ThT fluorescence was measured through the bottom of the plate every 30 min with a 450 nm excitation filter and a 480 nm emission filter in the absence of agitation. All measurements were performed in triplicate, and the experiment was repeated using at least two different protein batches. When required, aggregates were harvested from the reaction mixtures obtained in the absence of ThT by 100,000× *g* centrifugation for 1 h using an Optima Max Beckman ultracentrifuge (Beckman Coulter Inc., Brea, CA, USA). The obtained pellets were resuspended at 2 mg/mL in 25 mM Tris-HCl (pH 7.5) and 50 mM NaCl and stored at room temperature until use. For stability assays, aggregates at 0.04 and 0.2 mg/mL were dissolved in 0.1 M CaCl_2_ containing 10 μM ThT and the changes in the fluorescence of the amyloid probe were monitored for 20 h. The change in the ThT fluorescence was determined as % F = [(F_0_ − F_20_)/F_0_] × 100, where F_0_ and F_20_ are the ThT fluorescence intensities upon dilution and after 20 h incubation, respectively.

### 4.5. Dot-Blot Analysis

Aliquots containing 100 ng Ca^2+^-bound monomers and amyloids of Gad m 1 chains were spotted in duplicate on a nitrocellulose membrane. Immunodetection was performed by 1 h of incubation with anti-amyloid fibril OC (AB2286 Merck Millipore, 1/2000 dilution) and anti-amyloid oligomer A11 (AB9234 Merck Millipore, 1/2000 dilution) antibodies, followed by extensive washes and 30 min of incubation with horseradish peroxidase-labeled goat anti-rabbit IgG (1:5000 diluted; Sigma-Aldrich) [25,26]. ECL Western blotting reagent (BioRad) and a ChemiDoc XRS instrument (BioRad) were used for signal development and detection, respectively.

### 4.6. Atomic Force Microscopy

For AFM visualization, the products of the aggregation reactions were diluted 1/10 in 25 mM Tris-HCl (pH 7.5), 50 mM NaCl, and 4 mM EDTA. Typically, 30 μL of the resulting solutions was absorbed onto freshly cleaved mica via 5–10 min incubation at room temperature. The surfaces were then rinsed with double-distilled water and dried. Images were obtained using a MultiMode Veeco microscope with a NanoScope IIIa controller (NanoScope, Dallas, TX, USA) using rectangular cantilevers with tetrahedral tips (Oltespa, 2 N/m force constant and 70 kHz resonance frequency). Software to obtain and treat the images was provided with the instrumentation (NanoScope). AFM analysis was performed using the free program WSxM 4.0 (Nanotec, Madrid, Spain).

### 4.7. Calcium Carbonate Precipitation Experiments

Crystallization experiments were performed at 18 °C using sterile Lab-Tek chambers with permanox slides with covers (ThermoFisher Scientific, Waltham, MA, USA) inside a desiccator for CaCO_3_ crystal synthesis. Chambers were supplemented with 50 μL of 0.1 M CaCl_2_. For each experiment performed in duplicate, 1 and 5 μL samples of protein solutions (2 mg/mL) were added to the droplet. The chambers were sealed with parafilm, pierced with a needle, and placed in a desiccator. Constant CO_2_ vapor pressure for diffusion was generated by the decomposition of ammonium carbonate (25 mM) in 5 mL beakers. After 20 h, the crystals were rinsed first with Milli-Q (Millipore Corporation, Darmstadt, Germany) water and then with CaCl_2_-saturated methanol and allowed to air dry.

### 4.8. Microscopy Characterization of Calcium Carbonate Precipitates

Morphological information about the precipitates was assessed by SEM analysis using a Hitachi S-3000N scanning electron microscope (Hitachi, Tokyo, Japan) at 20 kV in the Microscopy Unit of the University Autónoma of Madrid. TEM imaging and SAED analysis were performed on a JEOL 300FEG electron microscope (JEOL, Peabody, MA, USA) operating at 300 kV in the ICTS-Microscopy National Center. To prevent beam-damage to the samples SAED patterns were obtained using low electron beam doses (beam intensities below 500 counts on the charge-coupled device (CCD) camera and an exposition time of 0.8 s).
